# Gestating times: women's accounts of the temporalities of pregnancies that end in abortion in England

**DOI:** 10.1111/1467-9566.12522

**Published:** 2016-12-02

**Authors:** Siân M. Beynon‐Jones

**Affiliations:** ^1^ Department of Sociology Wentworth College University of York York UK

**Keywords:** abortion, feminism, pregnancy, time

## Abstract

Tensions between the ‘clock time’ of medicine and the embodied times of its subjects are central to feminist writing concerning Western obstetric practice. In this article, I expand the focus of this literature by addressing the temporal dynamics of another site of reproductive healthcare: abortion provision. Echoing obstetric accounts of birth, time in legal, healthcare and social scientific discourse on abortion is routinely conceptualised as a finite resource contained within the pregnant/foetal body, which can be measured using clocks and calendars. I argue that women's interview accounts of their experiences of ending their pregnancies offer opportunities for critical reflection on this characterisation of pregnancy as linear ‘gestational time’. First, participants in this study re‐position the significance of gestational time by articulating its embodied meaning. Second, they provide alternative accounts of the temporality of pregnancy as a process which emerges through, and is disrupted by, the dynamics of socio‐material relations. The article considers the broader implications of women's accounts of pregnancy times for legal, healthcare and social scientific accounts of ‘later’ abortion.

## Introduction

Although it is constructed through specific practices of measurement, ‘clock time’ – the division of time into quantifiable, reproducible units – is routinely treated ‘*as* time’ (Adam 1995: 25 – emphasis added). Feminist scholarship critiques the hegemony of clock time within obstetric practice, highlighting the implications of this narrow temporal framework for women's experiences of birth (Adam 1995, Fox 1989, Martin 1989, McCourt 2009, Simonds 2002, Thomas 1992). Articulated in terms of the clock, time during birth becomes a finite resource to be managed (Adam 1995), as exemplified by the use of interventions to ‘speed up’ labours which transgress obstetric time‐frames (Pizzini 1992, Rosengren and DeVault 1963). Fox (1989) argues that this standardisation and objectification of time is alienating for women, for whom the temporality of birth is constituted by the particular lived experience of their contractions. Moreover, she suggests, it imposes an understanding of birth as a passive event whose passage can be traced in hours and minutes, as opposed to an active, time‐generating process through which women transform their own identities as they birth new life (see also Adam 1995).

In this article, I develop the insights of feminist scholarship concerning the temporal dynamics of medicalised childbirth, by addressing the temporalities of an alternative site of reproductive healthcare. Specifically, I explore women's interview accounts of their experiences with abortion and suggest that these afford opportunities to critically reflect upon available characterisations of pregnancy time. These include characterisations implicit within some of the feminist accounts of childbirth outlined above, namely, depictions of a time ‘contained within’ the rhythms of pregnant women's bodies.

## Conceptualising pregnancy times

As outlined above, feminist critiques of medicalised childbirth often centre upon conflicts between mechanised clock time and the variable rhythms of women's labouring bodies. However, as Adam (1995) notes, dichotomised accounts of time risk re‐producing a series of dualisms (objective vs. subjective; cultural vs. natural; linear vs. cyclical; mind vs. body) that are used to essentialise gender differences and inequalities. She advocates the exploration of the ‘simultaneity and mutual implication’ (Adam 1995: 6) of times and emphasises that, for a labouring woman, the rhythms of the body are experienced in relation to, and are inseparable from, the clock time which is central to social life in Western industrialised societies.

However, while Adam offers a non‐dichotomous accounts of temporal experience, she nonetheless approaches the times of ‘nature’ (i.e. bodily rhythms) as identifiable temporal realities which pre‐exist the socio‐material contexts in which they are embedded:

Birthing (without clinical intervention) is governed by internally organized rhythms that link hormone with muscle activity, synchronizing the needs of the unborn child with those of the mother's body. (Adam 1995: 48)

As a consequence, the ‘mutual implication’ of times that Adam considers seems to continue to assume an underlying ‘natural’ time, which proceeds independently of social relations. Arguably, this account of a time contained within the workings of the pregnant/foetal body *resembles* Western biomedical accounts of bodily time. Biomedicine assumes ‘an irreversible force of linear time in nature’ (Bledsoe 2002: 19) and, accordingly, conceptualises pregnancy as a biological process of foetal development, which unfolds automatically following the initiating event of conception (Bledsoe 2002, Franklin 1991, van der Sijpt 2012). The only (albeit, critical) difference between Adam's account, and this biomedical depiction of pregnancy time is that the latter insists it is possible to capture individually variable bodily rhythms using the chronologies of clocks and calendars. Pregnancies/foetuses are constructed in terms of ‘weeks’ of gestation using the date of a woman's last menstrual period or via standardised ultrasound measurements of foetal growth, which are correlated with units of clock time (Sänger 2015, Simonds 2002, Thomas 1992).

As I go on to highlight below, accounts of a time ‘contained within’^1^ the pregnant/foetal body have often been mobilised by those seeking to limit the provision of abortion to women. For this reason, in this article, rather than assuming a ‘natural’ time intrinsic to bodily processes, I conceptualise all time symmetrically as ‘created and sustained’ (Grabham 2014: 69) through particular socio‐material practices. In making this move I am indebted to insights provided by anthropological research, which has de‐naturalised and de‐stabilised Western assumptions of a time ‘in the body’ (Bledsoe 2002: 4). For example, Bledsoe (2002) demonstrates that, in the Gambia, the ageing and fertility of bodies is not dependent on linear biological processes which unfold autonomously in clock time. Rather, the rate at which a woman's body ages and her capacity to sustain a pregnancy and give birth are products of a ‘contingent’ (Bledsoe, 2002: 20) temporality of changing socio‐material relations (such as marriages, experiences of reproductive loss, or poverty). ‘Bodily resources erode in a highly saltatory manner, punctuated by dips, surges and lags […] the hardships of life inscribe themselves on the body at a pace that is contingent on external events’ (Bledsoe 2002: 322).

Focusing specifically on pregnancy, van der Sijpt (2012) illustrates a similarly socially ‘contingent’ understanding of bodily time in Cameroon. Biomedical models of pregnancy depict its outcome and meaning as knowable in advance, on the basis of increments of gestational time. In contrast, in Cameroon, the development of a pregnancy depends upon the strength of the blood of the parents, which in turn is mediated by socio‐material circumstances (such as the consumption of food, or physical labour). As a consequence, the outcome of a pregnancy (for example, whether a foetus is ‘viable’) is ‘contingent and unknowable, rather than a priori fixed trajectory’ (van der Sijpt 2012: 112). In this article I illustrate that women's accounts of abortion in England enable related re‐framings of the temporality of pregnancy, with implications for healthcare, legal and social scientific accounts of ‘later’ abortion**.**

## Gestational time in healthcare and legal accounts of ‘later’ abortion

Although its temporalities have received comparatively little attention, abortion provision represents a key context in which women are subject to particular characterisations of pregnancy time. Echoing obstetric accounts of birth, healthcare and legal discourse on abortion positions time as a finite resource contained within pregnant/foetal bodies, which can be measured using clocks and calendars. However, the significance of this ‘resource’ is expressed very differently across alternative discursive contexts. Healthcare discourse centres on the implications of gestational time for women's health, and for the improvement of abortion services. Weeks/days of gestation are used to predict the risks and efficacy of particular methods of abortion, with emphasis placed on avoiding the risks (and associated economic costs) that accumulate if ‘too much’ time passes before abortion. This approach is illustrated by the following extract from the Royal College of Obstetricians and Gynaecologists (2011) clinical guideline on *The Care of Women Requesting Induced Abortion* (hereafter, the RCOG *Guideline*):

An increase in the proportion of abortions performed under 10 weeks of gestation would result in significant cost savings for the NHS as a result of greater use of non‐surgical and local anaesthetic methods, as well as the reduced risks to women consequent to reduced gestation. Appointments should be expedited for women who present beyond 12 completed weeks of gestation […] to minimise further risk to health. (Royal College of Obstetricians and Gynaecologists 2011: 35)

In contrast, legal and public discourse on abortion in Scotland, England and Wales routinely focuses on the significance of gestational time for foetuses. The 1967 Abortion Act, as amended by the 1990 Human Fertilisation and Embryology Act, places an upper time limit of twenty‐four weeks’ gestation on most abortions (the procedure is permitted beyond this point, but on restricted grounds).^2^ This time limit was introduced in 1990 on the basis of medical arguments that twenty‐four weeks is the point in gestation beyond which it is possible to keep a foetus alive – with medical assistance – if it is born prematurely (Sheldon 1997). In recent years, claims about the capacities of later gestation foetuses have been mobilised as part of attempts to further lower this limit (Ingham *et al*. 2008, Palmer 2009). However, as Franklin (1991) highlights, the use of biological ‘viability’ to determine gestational time restrictions on the provision of abortion rests on a ‘teleological construction of the foetus’ (Franklin 1991: 200). Emphasis is placed ‘upon what the fetus is going to become, upon its genetically determined development’ (Franklin 1991: 197), as though the foetus contained its own adult future by virtue of the biological event of conception. This renders invisible the gendered bodily and social supports required to produce and sustain new persons (Franklin 1991, 1999, McNeil 1991). In so doing, it underscores the political implications of accounts which naturalise time as something ‘contained within’ the workings of the pregnant/foetal body.

In a previous study (Beynon‐Jones 2012), I illustrated how Scottish health professionals mobilise both ‘foetal‐centred’ (Steinberg 1991: 179) and woman‐centred representations of time and pregnancy to problematise the provision of ‘later’ abortions. Some health professionals argue that the passage of gestational time results in abortions which involve more ‘viable’ (and thus, more morally significant) foetuses, as well as more risks (both physical and emotional) for pregnant women. In problematising the accumulation of gestational time prior to abortion they also construct pregnancy as a temporal state of ‘inaction’, during which women ‘allow’ time to pass. However, other health professionals provide contrasting depictions of pregnancy as a ‘dynamic, potentially discontinuous process’ (Beynon‐Jones, 2012: 69) during which women's circumstances can change. They also re‐frame concerns about accumulating gestational time by emphasising the longer‐term alternative to later abortion, that is, forcing a woman to give birth and to pursue either motherhood or adoption. In this article I develop this work by illustrating how women's own accounts of abortion also broaden dominant temporal representations of pregnancy.

## Gestational time in research concerning ‘later’ abortion

Several studies within the UK (Ingham *et al*. 2008, Marie Stopes International 2005, Purcell *et al*. 2014) and the United States (Drey *et al*. 2006, Finer *et al*. 2006, Foster *et al*. 2008, Janiak *et al*. 2014, Kiley *et al*. 2010) have explored why women have abortions at ‘later’, rather than ‘earlier’, gestations. For example, Ingham *et al*. (2008) provide a quantitative analysis of the reasons for ‘delay’ which lead women in England and Wales to request an abortion in the second trimester of pregnancy. They suggest that, while barriers in service provision do play a role, ‘most reasons for delay are best considered “woman‐related”’ (Ingham *et al*. 2008: 27) in the sense that they take place prior to women's encounters with healthcare services. These primarily include difficulties involved in recognising pregnancy, concerns about the process of abortion, and difficulties involved in abortion decision‐making.

In a qualitative analysis of women's experiences of accessing abortion at ≥16 weeks’ gestation in Scotland, Purcell *et al*. (2014) similarly conclude that key ‘delays’ are generated by the difficulties involved in recognising pregnancy. Their study illustrates that women do not always experience ‘typical’ signs of pregnancy and that its recognition is also difficult when women do not expect to be pregnant (for example, because they are using contraception). It also highlights delays generated by the complexities of abortion decision‐making, including the regularity with which women experience major changes in their circumstances during pregnancy. In considering these issues, the authors draw attention to the agency which women have to exert in order to request a later abortion (Purcell *et al*. 2014).

This important body of research renders later abortion comprehensible by accounting for the passage of gestational time prior to women's requests for the procedure. Simultaneously, in exploring ‘why’ some women's abortions take place beyond particular gestations, it also begins from, and reproduces, two key assumptions that are central to healthcare and legal discourse. First, that pregnancy is a linear temporal event which unfolds automatically through clock time. Second, that the accumulation of gestational time prior to abortion is a problem of ‘delay’, which requires explanation (Beynon‐Jones 2012). As outlined above, the aim of this article is very different. It explores the ways in which women characterise the temporalities of pregnancies that end in abortion.

## Methods

This article draws on semi‐structured interviews with 28 women concerning their experiences of abortion. Seventeen women were recruited through clinics in England. Study information was provided to women by clinic staff on the day of their procedure, enabling them to contact me subsequently if they were interested in participating. However, this approach produced relatively few responses over a number of months. Reasons for this were unclear, but appeared to include difficulties in incorporating the study into busy clinic routines, as well as with the timing of the invitation to participate (staff suggested that women wanted to ‘move on’ from their experiences). Because of the slow rate of recruitment in clinics, 11 women were also recruited via adverts in newspapers and social media. All participants were offered a gift of £20 to thank them for their time. Ethical approval for the research was obtained from an NHS Research Ethics Committee, and the University of York's Economics, Law, Management, Politics and Sociology ethics committee.

Interviews were conducted either by phone or face‐to‐face (depending on participants’ preferences). They were recorded and professionally transcribed verbatim, except in one case where the recording device failed and detailed notes were written up immediately following the interview. All participants’ identities have been anonymised using an interview number. As I have explained elsewhere (Beynon‐Jones 2015), this decision was made because many women were anxious about concealing their identities and, following the interviews, I became concerned about the risks attached to employing pseudonyms (for example, if participants had hidden their ‘real’ names on their contact forms). Nonetheless, I remain troubled by the ways in which interview numbers de‐personify women's accounts. In any future research, I would approach this problem differently, by discussing the process of anonymisation with participants during interviews.

With the exception of one participant (who was English but had an abortion while living overseas), the experiences of abortion that women described all took place in England. There was considerable variation in the length of time that had passed since women's abortions (this ranged from approximately 3 weeks to 37 years), which means that participants had very different opportunities to reflect upon their experiences. However, in terms of the analysis presented in this article, time post‐abortion did not generate qualitative differences in the ways in which women talked about the temporalities of pregnancy.

Collectively, participants described experiences of ending pregnancies which ranged from approximately 5–22 weeks. To avoid imposing assumptions about the significance of gestational time during interviews, I tried to ask questions on this topic only in response to participants’ prior framings of its meaning. For example, if a woman said that she found it difficult being ‘so far along’, I asked her to tell me a bit more about why this was significant. In cases where it was unclear, I waited until the end of the interview before asking approximately how many weeks pregnant a participant was at the time of her abortion. Although this approach privileges a ‘gestational time’ account of pregnancy (van der Sijpt 2012), I asked for this information to ensure that the sample included the experiences of women who had ended their pregnancies at different gestations. I have also included it when presenting excerpts from the interviews because it underscores striking similarities between women's accounts, which arguably undermine the use of gestation as an automatic marker of difference.

Throughout this article, I have chosen to write about ‘abortion’, rather than employing the more clinical language of ‘termination of pregnancy’. This is because, while there are advantages to locating abortion primarily as a medical procedure, this approach can also marginalise discussion of pregnancy as a political issue of gender in/equality (Science and Technology Subgroup 1991, Sheldon 1997). At the same time, it is important to note that participants in the study drew on both terminologies, often interchangeably.

The decisions outlined above concerning the analytic language employed, and the presentation of participants’ accounts, underscore significant relations of power between myself and the women who took part in this study. While such relations could be said to be in flux during the research process (where, for example, participants can choose what to say), my role as ‘interpreter’ of the findings places me in a position of power (Letherby 2003). This position, in combination with the ongoing political contestation surrounding abortion provision in Britain, makes it crucial to emphasise that what follows is a ‘partial’ (Haraway 1991) depiction of women's narratives concerning abortion, produced through a specific (self‐selecting) process of recruitment and shaped by my particular analytic focus.

My analysis of the data explored how women characterised the temporal experience of pregnancies that end in abortion, and was facilitated by the qualitative data management software NVivo 10. As Bledsoe (2002: 19) highlights, the pervasive characterisation ‘of linear time in nature’ can make it analytically difficult to think outside of this framework, and pay attention to alternative formulations of time. Likewise, my initial coding of the interview transcripts focused solely on accounts of gestational time, and it was only on re‐reading the data that I realised participants were routinely describing pregnancy in other ways.

## Findings: pregnancy times

In the first section of the analysis I explore the ways in which, resonating with healthcare and legal discourse, women mobilised characterisations of gestational time as a finite resource when narrating their experiences of abortion. In the second section, I reveal how such ‘gestational time’ accounts were interwoven with an alternative depiction of the temporality of pregnancy.

### Embodying the ‘resource’ of gestational time

In describing gestational time as an ever‐diminishing resource, women both drew on, and complicated ‘teleological’ (Franklin 1991) accounts of autonomous foetal developmental time. This is because they described the accumulation of gestational time as a process that alters the relationships between pregnant women, their foetuses, and their bodies:

knowing that you're thirteen weeks and then you think obviously by the time the week after came I was nearly fourteen weeks and I thought obviously I'll do it now because if it had gone further on I wouldn't have done it […].^3^ Because three months and – at yeah fourteen weeks it's not fully developed. So I'd got used to that idea that it wasn't a fully developed baby. (Interview 26, 13 weeks’ gestation)

I said to my mum, ‘I don't want to obviously start getting any maternal feelings or anything now I know’ […] at the time I was really early on so in my head it was nothing, nothing really. And to wait another four weeks would have been a long time as in it would have been more of a baby, wouldn't it, by then. So that's why I think, you know, I was like, oh, shocked by the time I was going to have to wait. [*This participant went on to explain how she found a faster route to access the procedure than the one initially offered by her GP*] (Interview 21, 7 or 8 weeks’ gestation)

Another participant also described concerns about the socio‐material changes which gestational time could effect, arguing that 17 weeks was her personal ‘cut‐off point’ for having an abortion because of her fear that she might start to feel foetal movement at 18 weeks (Interview 3, 16 weeks’ gestation).

Elsewhere (Beynon‐Jones 2015) I have illustrated how women mobilise ultrasound measurements of gestation as ‘objective’ evidence of the moral/emotional insignificance of their own, early, pregnancy, contrasting this with imagined experiences of ‘later’ abortion. Other studies also draw attention to the value which some women attach to the gestational ‘earliness’ of abortion (Finer *et al*. 2006, Gerber 2002, Kirkman *et al*. 2011, Lattimer 1998). A key aspect of the findings presented here is that ‘earliness’ appears to be a relatively malleable resource which can be used to legitimate abortion across a wide range of gestations (e.g. at 7, 13, 16 weeks, etc.). However, it is also clear that this resource is not infinitely flexible. Several women spontaneously positioned their abortions as comparatively ‘late’, and described gestational time as a resource that they had lost. In several cases, this framework was used to explain difficult emotional experiences:

I mean, they can understand why I'm a bit upset because obviously I'd carried a baby and not really known it and it was fully – and it had all the stuff like the sex and everything that would have been able to distinguish. But to them [*the participants’ friends*,* who had experienced earlier abortions*] they just saw it as like a bunch of cells so it didn't really matter to them. It's still really nice that they can understand how I'm feeling about it and not be like, ‘Oh, it's just a bunch of cells I don't see why you care’. Which is the other response I get from some of my friends. When I get upset about it they'll go, ‘It was just a, it wasn't really a baby and it could have died anyway and like why do you care?’ (Interview 10, 18 weeks’ gestation)

The distress that this participant describes at others’ attempts to minimise the significance of her abortion underscores a critical point. While it is important to reflect upon the problematic implications of ‘gestational time’ accounts of foetal development, it remains vital to acknowledge the significance which this culturally specific understanding of pregnancy holds for some women (Morgan 1996).

In the accounts explored above, women articulate their own relationships with their pregnancies in terms of the passage of gestational time. In contrast, several participants focused on what it was like to encounter others’ framings of pregnancy as a process of ‘gestational time running out’. Women who requested abortion at above approximately 15 weeks’ gestation often described how legal and medical discourses positioned their pregnancies as gestational emergencies:

it all happened quite fast because obviously I was quite far gone and there's like the abortion cut‐off point is 24 weeks […] it was quite quick. They were good like in relation to like – because I was a bit scared that there might not be any appointments and that I'd have to keep it. (Interview 17, 22 weeks’ gestation)

at this point I'd only found out I was pregnant for about five days or so. So I was still digesting the whole situation myself […] And that was another issue that made me quite upset with it, is the timing. It was like you have to make your decision now – and that's it. But like it's one of those situations where that's how it is […] it's because it was so far along and they said if you need, if you are going to do it, you're safer to do it now.

Siân: Right, ok. So safer in terms of risks to you and stuff or?

Yeah. Because they said I was in quite a risky like – I was quite far along so they said it is still quite risky but if you – the further along you are the riskier it is. And when you get to 24 weeks its, sometimes they don't even do it. (Interview 16, 15 weeks’ gestation)

While for many participants, speed was valued, some – such as Interviewee 16 – described the compression of time as an additional source of stress, which made it impossible to process the events that had happened to them.

As well as drawing attention to the significance of the legal time limit on abortion, Interviewee 16 highlights what it is like to be positioned as someone whose body has accumulated, and is continuing to accumulate, ‘risks’ through the passage of gestational time. She accepts that this is simply ‘how it is’, and articulates her frustration with her situation, rather than criticising the way in which it was framed by health professionals. When compared with earlier abortion, the risks of later abortion become higher and – as the RCOG *Guideline* (2011) points out – health professionals have an obligation to inform women of the risks associated with different procedures. However, it is also possible to contextualise the risks of later abortion by emphasising that, ‘in a legal setting where sterile facilities are available, abortion is a safe procedure for which major complications and mortality are rare at all gestations’ (Royal College of Obstetricians and Gynaecologists 2011: 1). One participant described how an encounter with this alternative depiction of all abortions as relatively ‘safe’ across gestational time normalised having a later gestation procedure:

And actually when you look – they give you a little book and when you look at the figure for like risks they're not dissimilar to an early termination. And that's what the lady pointed out to me in [*clinic location*] and she said to me you'll be absolutely fine. (Interview 24, 16 weeks’ gestation).

### Gestating times: beyond a time ‘contained within’ the pregnant/foetal body

Women's accounts of gestational time were interwoven with an alternative depiction of the temporality of pregnancy, which I have termed ‘gestating times’. Below, I suggest that, although it is produced within a completely different cultural context, this depiction resonates in key ways with Bledsoe's (2002) analysis of ‘contingent’ bodily temporalities in the Gambia, as well as van der Sijpt's (2012) account of pregnancies in Cameroon. This is because, in describing their experiences of abortion in England, women also offer an understanding of pregnancy as dependent upon the unpredictable rhythms of context‐specific socio‐material relations.

### Becoming pregnant

In opening interview conversations with participants, I asked them if they could ‘tell me a bit about what happened when you first thought that you might be pregnant?’ In response, women tended to provide lengthy narratives of events that seemed to take place long before the subject of my question:

I actually managed to conceive at the beginning of July this year but didn't find out until around the end of August – I think around the beginning of September. It might even have been mid‐September actually. And I was, I stupidly did make the mistake of relying on the after‐effects of being on the injection because it does affect your fertility. And I'd, I'd basically I made that mistake because I hadn't had a period in a good six months. [*Later in the interview*,* the participant explained that the contraceptive injection had stopped her periods*. *When they didn't resume after the effective period of the injection had ended she assumed that it had continued to affect her fertility]*. And so I took the morning‐after‐pill just to be on the safe side and I had a period like straight after. So I thought, well okay, that's usually a *pretty* good sign that you haven't conceived. I went on holiday for a week, a drinking holiday, which I feel terrible about now and went about my normal life. And I took a test just in case because I wanted to go on the Pill because I'd started seeing someone properly, and this is the same person. So I was like I need to make sure that I'm on some proper contraception not just using condoms and stuff like that. […] So basically I took a test to be sure that I definitely wasn't before I started going on the Pill and it was negative […] so the doctor basically gave me the all clear to go on the Pill. I went on the Pill for twenty‐one days and it came to my seven day break and nothing happened. So I was like, ‘Oh this can't be good’. Maybe it's just my body being funny because, as I said, I'd been used to not having periods for nearly three years. And so I took a test at work – and it came up positive and I was like, ‘What?’ (Interview 7, 9 weeks’ gestation)

This account can be understood as a retrospective justification for pregnancy produced within the confines of discourses which insist that contraception gives women complete control over the timing of conception (Beynon‐Jones 2013, Ruhl 2002, Thomas 1992), and that the passage of gestational time prior to abortion requires explanation. However, this process of retrospective accounting simultaneously depicts the temporalities of conception and pregnancy in ways that challenge dominant narratives. Although this participant begins by trying to locate conception as single ‘initiating’ event in clock time, she goes on to describe the process of ‘becoming pregnant’ as a bodily state that emerges over an extended time period, in relation to multiple, inextricable, socio‐material processes with varying temporal rhythms. Pregnancy is produced through the interplay between the dynamics of heterosexual relationships (the transition to ‘seeing someone properly’ and its implications for contraceptive practice), and the disappearance and reappearance of bleeding in relation to multiple reproductive technologies (the contraceptive injection, the pill, the morning after pill and two pregnancy tests).

A striking (and counter‐intuitive) feature of this account is the extent to which hormonal contraceptive use is described as central to the process of *becoming* pregnant. This issue emerged in several interviews:

I was on the contraceptive Pill, I'd been on it for four months a new brand […] and I hadn't had a period at all, from being on it. I took a pregnancy test a month after starting it and it was negative and the woman just told me that I didn't get periods on it and I should probably just go about it as if it was normal. And then when my friend saw me she said, ‘God, you've put on a lot of weight!’ And I hadn't realised. […] and she kind of joked and said, ‘Are you sure you're not pregnant?’ And I was like, ‘No, there's no way I could be’. And then we went out a couple of nights and every time I came back in the morning I'd be like really, really horribly sick. And I hadn't had any kind of morning sickness or anything like that. I'd had the sore boobs and everything but I just thought that was probably because I was due on kind of in my Pill kind of thing but not actually having a period. And then […] she was like ‘I really think you need to have a pregnancy test’ […] I did two of them at the same time and they were both really, really strongly positive and I started crying. (Interview 10, 18 weeks’ gestation)

Participants described using hormonal contraception as an experience of temporal adaptation through which they learned to live with new expectations about the symptoms of their bodies (for example, particular patterns of bleeding, or periodically sore breasts). Their accounts focus on the labour involved in the transition from non‐pregnant to pregnant embodiment, a process which requires learned bodily practices of infertility to be transformed into an experience of fertility. They also depict the ‘moment’ of transformation as one of shocking bodily disruption and temporal dislocation. Related accounts were offered by women who were not using hormonal contraception but likewise experienced their bodies as infertile, for example, because they bled regularly, or had sex infrequently.

For several women, ultrasound dating prior to abortion became an additional site of temporal dislocation. Through ultrasound scans, lived experiences of pregnant embodiment were brought into collision, and rendered completely asynchronous with, medical measurements of gestational time:

And then she told me and said, ‘Do you want to know how far along you are?’ and I said, ‘Yeah’. And she said that I was 17 and a half weeks and I just burst out into tears and so did my boyfriend. He just burst out into tears. We couldn't believe it was that far along without even knowing and not really even showing. Because I still got quite a, I still had quite a flat belly then. (Interview 10)

I went to the ultrasound clinic and basically had a scan. And I had a look and everything like that. And I can remember them just saying to me, ‘Oh yeah, you're 15 weeks’, and I was just like, ‘What?’ I literally, I couldn't comprehend it in my mind that it was *that* long. (Interview 16)

In these extracts, in spite of the shock with which they are received, ultrasound measurements of gestation are treated as largely authoritative accounts of the temporality of pregnancy, with which women have to reconcile their bodily experiences.

### Making contingent futures

Lattimer (1998) suggests that accounting for abortion decisions requires women to reconcile conflicting social norms. On the one hand, motherhood is portrayed as the correct outcome of pregnancy for all women. On the other, it is depicted as an event that should occur at the correct time in a woman's life. Accounts of abortion decision‐making offered by women interviewed for this study can likewise be read in relation to dominant discourses concerning femininity and motherhood within a particular social context. However, my argument in this article is that they also offer a particular characterisation of the temporality of pregnancy, as a process of making socio‐materially contingent futures:

I was far gone with my second one. I think I was about 15 or 16 weeks. And that was because I had booked an appointment, then changed my mind, and then the domestic violence happened again and I said no, I'm going to book it again. (Interview 13, 15 or 16 weeks’ gestation)

I didn't find out I was pregnant till I was 21 weeks. Because I was still having periods and I wasn't being sick or anything like that. And then I found out on the [*particular day*] and that [*same*] night my partner got laid off work so then it just made the choice easier just to have the termination. […] He wanted – he actually wanted to keep it. We had a lot of heated discussions but then he came to his senses basically and said well we can't really afford to have it. (Interview 1, 22 weeks’ gestation)

Such accounts of the ways in which pregnancy and abortion engender particular futures are far removed from the ‘teleological construction of the fetus’ (Franklin 1991: 200) central to legal debate about gestational time limits. The first participant describes how the viability/non‐viability of her pregnancy fluctuated in relation to the episodic violence to which she was subjected by her partner. The second describes the long‐term viability of her pregnancy as dependent upon the economic support required to sustain child‐rearing, highlighting how the discovery of her pregnancy coincided with the loss of such support.

### Inextricable pregnancy times

It is clear that women did not treat ‘gestational time’ and ‘gestating times’ as mutually exclusive accounts of pregnancy. Rather, their narratives draw upon both versions (for example, talking about being ‘quite far gone’, whilst highlighting the contingent contexts which make it possible for pregnancies to materialise particular kinds of future). This ‘mutual implication’ (Adam 1995: 6) of times is particularly striking in the following example, in which a participant describes attending her GP to seek an explanation for a bout of sickness and being told, following an ultrasound, that she was 20 weeks pregnant:

I went from, you know, one day thinking, oh, I'm being sick quite a lot, this is a bit weird to the next being like four months pregnant, or five. Like it sort of, it put it into perspective a lot more. I just think – I don't really know because I haven't experienced it the other way round – but like it became very real very quickly. Like whereas if I'd found out when I was like three weeks and then had known about it for a long time and sort of gotten used to it and whatever and then at that stage had been five months pregnant I don't think that I would have been able to follow through with a termination in that situation.

Siân: Can you sort of say a bit more about what you mean by that. When you were saying it was sort of, it was real very quickly?

Like I hadn't really got time – I hadn't gotten used to the idea of, oh these are the life changes that I could make and I've got a long, it's all right because I've got a long time to do them, to get everything sorted out or anything like that. It was sort of like – instead of, instead of like imagining how I could change my life for a baby to fit in it was me sort of looking at my life *now* thinking a baby couldn't – like I could *not* bring a baby into this environment. (Interview 25, 22 weeks’ gestation)

This participant describes the social sustainability of her pregnancy as entangled with its location in gestational time. The transformation from ‘being sick quite a lot’ one day, to being five months pregnant the next day, she insists, is different from living with a pregnancy for five months. She suggests that the loss of the resource of gestational time truncated the time‐frame of her pregnancy, producing a stark confrontation with its lack of socio‐material viability.

## Conclusion

This article extends existing analyses of time and reproductive medicine by exploring women's characterisations of the temporalities of pregnancies that end in abortion. A key finding is that many participants described their pregnancies in terms of the finite, and rapidly‐diminishing resource of ‘gestational time’. However, while their accounts reproduce elements of healthcare and legal discourse concerning abortion, they also articulate a subject position which is routinely missing from this discourse. Specifically, they describe gestational time as an embodied resource, making it possible to consider what it might mean to be a pregnant woman whose capacity to enact reproductive decisions is threatened by the prospect of ‘time running out’. Women routinely talked about the accumulation of gestational time as a threat to their agency and bodily autonomy. In some cases, this threat was described in terms of fears about the development of increasingly significant bodily relationships between a pregnant women and her foetus. In other cases, it was described as the product of encounters with healthcare services, and the experience of being positioned as a gestational emergency.

The analysis suggests that there might be ways in which health professionals could normalise the passage of gestational time and mitigate the experience of ‘time running out’, in particular, by contextualising the comparative ‘riskiness’ of later abortion. Nonetheless, there are clear constraints on the possibilities of practice in this field of healthcare. In particular, the small number of clinics which provide later gestation procedures (Lee *et al*. 2004, Purcell *et al*. 2014), and the upper time limit in abortion law means that ‘time running out’ forms part of the framework within which health professionals have to work.

I have argued that, interwoven with the depiction of pregnancy time as a finite resource contained within the body, participants also provided an alternative account of the temporality of pregnancy. Women suggested that pregnancy emerges from, and is sustained by, a complex interplay of multiple socio‐material relations with varying temporal rhythms (for example, the appearance/disappearance of menstrual symptoms in relation to the gendered burden of contraception, the shifting dynamics of heterosexual relationships, and changes in paid work). In contrast to portrayals of pregnancy as unfolding through linear time following the ‘event’ of conception, such accounts foreground the multiple agencies (human and non‐human) involved in producing, recognising and sustaining a pregnancy, and the discontinuities and disruptions entailed in these processes.

Accounts of pregnancy as a contingent process of ‘gestating times’ pose an important challenge to the ‘teleological’ (Franklin 1991: 200) depictions of foetal development which have become so central to the discussion of legal time limits on abortion. They also offer an alternative to narratives of later abortion as a problem of ‘delay’, raising questions about the centrality of this narrative within policy, practice and research concerning abortion. Does an emphasis on ‘delayed’, accumulated, weeks of pregnancy leave space in which to discuss pregnancy as a process without a clear ‘beginning’, produced through cumulative, distributed agencies, including those (e.g. hormonal contraceptives) employed in its prevention? Similarly, does it facilitate understandings of pregnancy as dependent upon sudden, unpredictable fluctuations in the relations which sustain it? Finally, does it enable articulations of pregnancy as an experience of shocking, traumatic, temporal dislocation?

A focus on ‘gestating times’ foregrounds, and makes it possible to problematise, the gendered socio‐material relations of pregnancy, rather than the passage of clock time. Nonetheless, the analysis presented here has emphasised that women describe pregnancy as the product of ever‐changing socio‐material relations *and* as weeks of ‘gestational time’. Furthermore, as indicated in the introduction, gestational time has long been harnessed by clinicians as a method of measuring and standardising pregnant bodies in attempt to research and improve abortion provision. Accordingly, rather than displacing a ‘gestational time’ account of pregnancy, this article has problematised its reification as the only basis from which to reckon pregnancy time(s). In doing so, it has drawn attention to the limitations of the singular account of the temporality of pregnancy that dominates contemporary public discussions of abortion in England.

## Notes

1 My use of the notion of a time ‘contained within’ the pregnant/fetal body draws directly on Bledsoe's conceptualisation of a ‘chronological “time in the body”’ (Bledsoe 2002: 4) as the dominant Western trope for describing the temporality of bodily processes. However, I employ this expression in a more expansive sense. Rather than simply capturing accounts of bodily time as synonymous with clocks, I use it to refer to any account in which time is naturalised as intrinsic to bodily processes (for example, as in Adam's depiction of the organic rhythms of the labouring pregnant body).

2 Regardless of gestation, abortion requires the signed agreement of two doctors.

3 Note on transcription: […] denotes omitted text. [*Italicised text in brackets is my annotation*]
